# Complete Genome Sequence of the Type Strain Aliivibrio fischeri DSM 507

**DOI:** 10.1128/mra.00801-22

**Published:** 2022-11-10

**Authors:** Kalliopi-Konstantina Papaioannou, Jacqueline Hollensteiner, Judith Katharina Helena Witte, Anja Poehlein, Rolf Daniel

**Affiliations:** a Genomic and Applied Microbiology and Göttingen Genomics Laboratory, Institute of Microbiology and Genetics, Georg-August University of Göttingen, Göttingen, Germany; Indiana University, Bloomington

## Abstract

The complete genome sequence of the type strain Aliivibrio fischeri comb. nov. DSM 507 is presented. The genome consists of two circular chromosomes comprising 2,970,859 bp (chromosome 1) and 1,532,347 bp (chromosome 2) with GC contents of 38.94 and 37.13%, respectively.

## ANNOUNCEMENT

The genus *Aliivibrio* comprises Gram-negative, rod-shaped gammaproteobacteria, which are abundant in marine environments (1). Aliivibrio fischeri was first described for bioluminescence (2) and served as model for bacterium-host interactions (3). In total, 81 A. fischeri genomes were available at the NCBI database (accessed 27 July 2022) previously, of which 1 was closed. The type strain A. fischeri DSM 507 genome was in a draft status.

A. fischeri DSM 507^T^ (ATCC 7744^T^ = CAIM 329^T^ = CCUG 13450^T^ = CIP 103206^T^ = LMG 4414^T^ = NCIMB 1281^T^) was obtained from the German Collection of Microorganisms and Cell Cultures GmbH (DSMZ; Braunschweig, Germany). It was first described in 1889 by Beijerinck as Photobacterium fischeri (4). For DNA extraction, the strain was cultivated in Bacto marine broth (BD Difco marine agar 2216; Thermo Fisher Scientific, MA, USA) at 22°C with shaking for 3 days. Genomic DNA was extracted using the MasterPure complete DNA purification kit (Epicentre, Madison, WI, USA) according to the manufacturer. DNA was sequenced using both Illumina (Illumina, San Diego, CA, USA) and Oxford Nanopore (Oxford Nanopore Technologies, Oxford, UK) technologies. Illumina paired-end libraries were constructed using the Nextera XT DNA sample preparation kit and sequenced using a MiSeq instrument and reagent kit v3 (600 cycles, 2 × 300 bp) as recommended by the manufacturer (Illumina). The Nanopore DNA library was prepared by using unsheared DNA without size selection by employing the ligation sequencing kit 1D (SQK-LSK109) and the native barcode expansion kit (EXP-NBD114; barcode 17) as described by the manufacturer (Oxford Nanopore Technologies). The MinION device Mk1B, with an R9.4.1 SpotON flow cell, and MinKNOW software v21.10.4 were used (72 h; Oxford Nanopore Technologies). For demultiplexing and base-calling, Guppy v6.0.1 (Oxford Nanopore Technologies) was applied with high-accuracy mode. Quality control was performed with fastqc v0.11.9 (5). Default parameters were used for all software unless otherwise stated. Sequencing resulted in 3,296,302 Illumina reads and 61,342 Nanopore reads (*N*_50_/*N*_90_, 10,839/2,305). Illumina and Nanopore reads were quality filtered and adaptors were removed with fastp v0.23.1 (6) and Porechop v0.2.4 (https://github.com/rrwick/Porechop.git; accessed March 2022). A *de novo* hybrid assembly was performed using Unicycler v0.4.9 in normal mode (7). Quality was inspected with Bandage v0.8.1 (8). Coverages were calculated with Qualimap v2.22-r1101 (9) using Bowtie2 v2.4.4 (10) and Minimap2 v2.22 (11). PGAP v6.0 (12) was used for genome annotation. The assembly revealed two circular chromosomes comprising 2,970,859 bp (chromosome 1) and 1,532,347 bp (chromosome 2) (GC contents, 38.94 and 37.13%, respectively) with an overall mean coverage of 239-fold. The genome harbored 4,084 putative genes, of which 3,926 were protein encoding. Additionally, genes encoding 123 tRNAs, 31 rRNAs, 1 transfer-messenger RNA (tmRNA), and 3 noncoding RNAs (ncRNAs) were identified.

**FIG 1:**
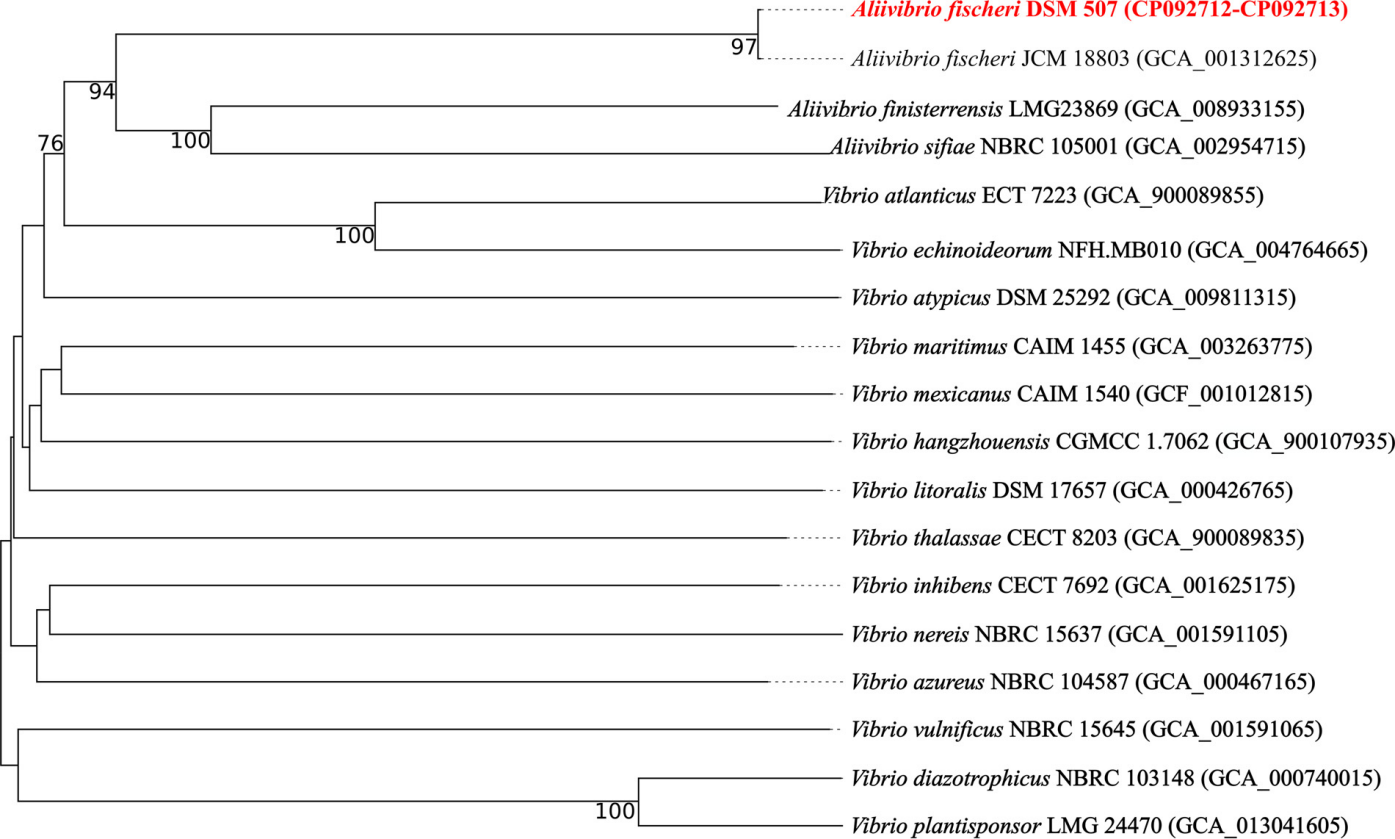
Whole-genome-based phylogenetic classification of Aliivibrio fischeri DSM 507. The genome BLAST distance phylogeny (GBDP) tree was generated with the Type Strain Genome Server (TYGS [13]; accessed 6 March 2022). The tree was inferred with FastME 2.1.6.1 (14) from GBDP distances calculated from genome sequences. The branch lengths are scaled in terms of GBDP distance formula d_5_. The numbers at the nodes are GBDP pseudobootstrap support values of >60% from 100 replications. The average branch support was 52.8%. The tree was midpoint rooted (15).

Whole-genome-based phylogeny of the DSM 507^T^ genome was performed using the Type (Strain) Genome Server (TYGS) (13). The highest similarity detected was to the draft type strain A. fischeri JCM 18803 genome (Assembly accession number GCA_001312625.1; 180 contigs). Aliivibrio finisterrensis LMG 23869^T^ (GCA_008933155.1) and Aliivibrio sifiae NBRC 105001^T^ (GCA_002954715.1) were the closest relatives (Fig. 1), with similarities of 25.7% and 23.6% (dDDH-d_4_), respectively.

### Data availability.

The complete genome sequence is available at DDBJ/ENA/GenBank under the accession numbers CP092712 (chromosome 1) and CP092713 (chromosome 2). The raw reads were deposited in the NCBI Sequence Read Archive (SRA) under the accession numbers SRR18969867 (Illumina) and SRR18969866 (Nanopore).
